# Knowledge and Attitudes towards Vitamin D among Health Educators in Public Schools in Jeddah, Saudi Arabia: A Cross-Sectional Study

**DOI:** 10.3390/healthcare10122358

**Published:** 2022-11-24

**Authors:** Amal S. Hamhoum, Najlaa M. Aljefree

**Affiliations:** Food and Nutrition Department, Faculty of Human Sciences and Design, King Abdulaziz University, Building 43, Room 237, Level 2, Jeddah 3270, Saudi Arabia

**Keywords:** vitamin D, knowledge, attitude, health educator, public schools

## Abstract

Health educators in schools are a very important part of the education system. Considering the significant prevalence of vitamin D deficiency in the Kingdom of Saudi Arabia, it is important to investigate the knowledge of and attitude towards vitamin D among health educators. This study aims to examine the knowledge of and attitude towards vitamin D among health educators in public schools in Jeddah as well as to identify the associated sociodemographic factors. A cross-sectional study was conducted between May and December 2021 among 231 health educators. Data were collected via a self-administered online questionnaire. The results revealed that only 45% of health educators had good knowledge of vitamin D, and approximately 43% had a positive attitude towards vitamin D. Additionally, those who had good knowledge of vitamin D were males (58.7%) (*p* = 0.005) and had a bachelor’s degree (74%) (*p* = 0.01). Moreover, male health educators aged 45–54 years had a positive attitude towards vitamin D (3.8 ± 0.7) (*p* = 0.007). In addition, female health educators who were divorced (3.8 ± 0.7) and widowed (3.6 ± 0.5) (*p* = 0.04) and those who were administrators (3.3 ± 0.7) (*p* = 0.01) had a positive attitude towards vitamin D. The Ministry of Education (MOE) in the Kingdom must educate health educators through educational programmes that aim to increase the knowledge of and develop a positive attitude towards vitamin D intake.

## 1. Introduction

Vitamin D deficiency is a key public health concern worldwide. Recent studies have revealed the importance of vitamin D in maintaining good health and preventing numerous diseases [1]. Although the main role of vitamin D is to maintain bone and skeletal health and prevent rickets and osteoporosis [2], it also plays a vital role in other non-skeletal systems of the body, such as strengthening the immune system and relieving the severity of chronic diseases such as cardiovascular disease, high blood pressure, and diabetes [3,4]. The recent literature has revealed that vitamin D has an impact on the mental health of children and prevents the exacerbation of mental health problems [5]; it also plays a role in the prevention of obesity [6] and sleep disorders [7] among children and adolescents. Moreover, several studies have indicated the role of vitamin D in reducing mortality due to numerous chronic diseases, including cardiovascular disease, cancer, and respiratory diseases [4]. Various scientific studies have revealed widespread vitamin D deficiency among all age groups worldwide [8,9,10,11,12,13]. In the United States, the incidence of vitamin D deficiency (<50 nmol/L) is reported to be 39% among the general population [8]. In northern Europe, the prevalence of vitamin D deficiency (<50 nmol/L) ranges from 6.6% to 33.6% in adults [9], while the prevalence of vitamin D deficiency (<50 nmol/L) among adolescents in Norway and Denmark is 39% and 51%, respectively [10,11]. In the United Kingdom (UK), the prevalence of vitamin D deficiency (<50 nmol/L) among the adult population is 61.4% [12]. In Asia, almost half of the Chinese, Indian, and Malay adults who participated in a study that measured vitamin D levels had vitamin D deficiency (<30 ng/mL) [13]. In addition, vitamin D deficiency is highly prevalent in Saudi Arabia as well [14]. A study reported vitamin D deficiency (<50 nmol/L) levels of up to 40.6% among males, 62.7% among females, and 50% among pregnant and lactating women [15]. In addition, research conducted in Jeddah in Saudi Arabia reported that 67.5% of Saudi adolescent girls experienced vitamin D deficiency (<50 nmol/L), which makes them more vulnerable to developing issues related to vitamin D deficiency, such as osteoporosis [16]. Furthermore, a high burden of vitamin D deficiency (≤25 nmol/L) was reported among children in Saudi Arabia, reaching 93–98% [17]; moreover, approximately 45.7% of the elderly were reported to have a vitamin D deficiency (<50 nmol/L) [18]. Similarly, a study conducted among children up to the age of two years in the Asir region of Saudi Arabia indicated that vitamin D deficiency (<20 ng/mL) was prevalent in 50% of these children [19]. The majority of studies linked to vitamin D deficiency recommend providing health education to the groups that are most vulnerable to vitamin D deficiency, such as females, children, and female adolescents [20].

Health educators in schools are a very important part of the education system due to their direct contact with children in schools at an early age; these educators play an important role in establishing a strong knowledge of health among future generations. The Ministry of Education (MOE) in Saudi Arabia has appointed health educators for all schools. The role of these health educators is to provide the necessary health education for young students and to teach them the concepts and impart them with the knowledge required to develop the value of health and well-being among future generations [21].

Additionally, health educators are in contact with teachers and parents and, thus, extend their impact to the entire community. Considering the significant occurrence of vitamin D deficiency in the Kingdom, the scarcity of knowledge of and negative attitudes towards the health benefits of vitamin D and its sources might be the reason for the high prevalence of vitamin D deficiency. In addition, only a few studies in the extant literature have examined the knowledge of and attitudes towards vitamin D in Saudi Arabia [22,23,24]. Part of the responsibility for disseminating knowledge and developing a positive attitude towards vitamin D in the community rests with health educators in schools; good dissemination of such knowledge and the development of a positive attitude will likely lead to an enhanced level of community health and reduced risk of vitamin D deficiency in the country’s population. Furthermore, it is vital to investigate health educators’ knowledge of, and attitude towards vitamin D deficiency within schools, where a large group of individuals are at risk of vitamin D deficiency and are those who require health education programmes. In fact, health educators in schools are neglected in terms of scientific research and health interventions. Hence, the current study aims to fill this gap and examine the knowledge of and attitude towards vitamin D among health educators in public schools in Jeddah, Saudi Arabia, and to identify the associated sociodemographic factors.

## 2. Methods

### 2.1. Study Design and Participants

A cross-sectional study design was employed for this study, which was conducted in Jeddah between May and December 2021. Ethical approval was obtained from the Biomedical Ethics Research Committee of King Abdul-Aziz University (Reference No. 128-21). Ethical approval was also obtained from the Department of Education in Jeddah. All participants were health educators who worked in different stages of public schools (primary, intermediate, and secondary) for boys and girls. According to the statistics from the Ministry of Education (MOE), public schools in the city of Jeddah belong to one of four educational administrative offices for girls (North, Central, East, and South) and six educational administrative offices for boys (North, East, Central, Naseem, South, and Safa). The number of public schools for boys was 507, including 243 primary schools, 164 middle schools, and 100 secondary schools [25]. The number of public schools for girls was 557, including 255 primary schools, 172 middle schools, and 125 secondary schools [25]. There was at least one health educator in each school and, thus, there were approximately 1064 health educators working in public schools in Jeddah. By using an online sample size calculator, the sample size required for this study was 216 health educators [26].

The inclusion criteria for study participants included adults (18 years old and above) from both genders who work as health educators in public schools in Jeddah city. The exclusion criteria included health educators who work in private schools. Two hundred and thirty-one (112 male and 119 female) health educators were involved in this study. The majority of these health educators were from East Jeddah (32%) and North Jeddah (25.5%), followed by South Jeddah (18.2%) and Centre (15.6%). The lowest percentage of participants came from the administrative offices of Asafa (3.9%) and Naseem (4.8%).

### 2.2. Data Collection

The data collection was conducted through a self-administered online questionnaire that was sent to the six above-mentioned educational administrative offices. Subsequently, the online questionnaire was distributed among the schools in each administrative area. The online questionnaire had a short introduction explaining the research goal and emphasized that participation was voluntary and anonymous. Additionally, it explained that completion of the questionnaire indicates accepting participation in the study. Questions regarding knowledge and attitude towards vitamin D were adapted from previous validated questionnaires [27,28,29] which were originally written in English. Consequently, the questionnaire was translated into Arabic and then translated back into English. Furthermore, to ensure the validity of the questionnaire, three nutrition scholars had included comments in the questionnaire to ensure that the included questions would be easily understood by potential respondents. In order to maintain reliability, the questionnaire was piloted among 10 health educators to guarantee that all questions were adequately understood. The questionnaire comprised three sections: sociodemographic factors of the health educator (gender, age, marital status, qualification, school stage, specialization, and years of experience), participant’s knowledge of vitamin D (consisting of eleven questions), and participant’s attitude towards vitamin D (consisting of five questions). 

### 2.3. Knowledge and Attitudes Scores towards Vitamin D

With regard to knowledge of vitamin D, participants were asked whether they had heard of vitamin D and whether they believed vitamin D was important for their health. They were also asked about their source of information on vitamin D and where they believe the body acquires the vitamin from. The questions also included the food sources rich in vitamin D, benefits of vitamin D, people at risk of developing vitamin D deficiency, factors affecting the synthesis of vitamin D in the human body, vitamin D and calcium absorption, and vitamin D supplements (Supplementary Materials). To consider health educators as knowledge-competent and as having positive attitudes towards vitamin D, they had to score over 50% in the total knowledge and attitude scores. In knowledge scores, question number 1 had two possible answers and was given one point for the correct answer and zero points for the incorrect answer. Question number 2 and questions 4–11 had multiple correct answers and were awarded one point for each correct answer and zero points for an incorrect answer. Moreover, the overall knowledge scores ranged from 0 to 25; knowledge scores in the range of 0–13 were categorized as poor knowledge and knowledge scores in the range of 13–25 were categorized as good knowledge. 

For questions pertaining to the attitudes towards vitamin D questions, participants were asked whether they like to expose themselves to sunlight, believe vitamin D is important for health, and agree that exposure to sunlight is harmful to the skin. They were also asked whether they were concerned that their current vitamin D levels might be too low and whether they were willing to undergo tests for determining their vitamin D levels. Questions 1–4 had three possible responses: ‘Agree’, ‘Disagree’, and ‘Neither agree nor disagree’. The response ‘agree’ was given one point, and the other responses were awarded zero points. Additionally, question 5 had only two responses: ‘yes’ and ‘no’. The response ‘yes’ was awarded one point, and the response ‘no’ was awarded zero points; thus, attitude scores ranged from 0 to 5. These attitude scores were categorized as negative attitudes (0–3) and positive attitudes (3–5).

### 2.4. Statistical Analyses

All statistical analyses were performed using SPSS statistical analysis software (version 22). The sociodemographic characteristics and answers pertaining to the knowledge of and attitude towards vitamin D are presented as frequencies and percentages. Additionally, scores pertaining to the knowledge of and attitude towards vitamin D are presented as mean ± SD. The chi-square test was used to examine associations between sociodemographic characteristics and knowledge of and attitude towards vitamin D. Furthermore, a linear regression was conducted to examine the association between sociodemographic characteristics and knowledge of and attitude towards vitamin D scores. A *p*-value of 0.05 was considered statistically significant.

## 3. Results

### 3.1. Sociodemographic Characteristics

The sociodemographic characteristics of health educators classified by gender are presented in Table 1. The study included 231 health educators, of which 48.5% were males and 51.5% were females. Additionally, 60% of health educators were in the age group 35–44 years (61.6% male and 58% female), and almost 40% were aged 45 years and over (19.7% male and 37.8% female). The majority of health educators in the study were married (84%), held a bachelor’s certificate (69%), had 6–10 years of work experience (74%), were administrators (73%), were working in primary schools (44%), and were from the North (25.5%) and East (32%) educational administrative offices in Jeddah. Furthermore, the health educators’ educational backgrounds were Science (23%), Islamic Studies (15.6%), Linguistic Studies (15.6%), and Social Science (13.4%). There were significant gender differences in the health educators’ age, marital status, work experience, and school stage. Male health educators were younger (*p* < 0.001) and had longer work experience (*p* < 0.001) than female health educators; moreover, male health educators more commonly worked in secondary schools (*p* < 0.001). The highest percentage of health educators from both genders were married, the proportion of single males was higher than that of females, and the proportion of divorced and widowed females was higher than that of males (*p* = 0.05).

### 3.2. Knowledge Regarding Vitamin D

Health educators’ knowledge regarding vitamin D questions and responses are presented in Table 2.

Almost all of the participants were aware of vitamin D and believed it is important for their health. Moreover, 75% of health educators knew the sources of vitamin D, including diet, sun exposure, and supplements. Additionally, 70% of participants knew that fatty fish (salmon and sardines) are a good source of vitamin D, while one-third knew that milk and eggs are a good source of vitamin D. However, a few participants recognized vegetables and fruits (30%) and olive oil (10%) as good sources for the vitamin. Additionally, 94% of health educators were aware that vitamin D is important for bone health, and 27% were aware that it can prevent heart diseases. Additionally, the majority of participants correctly identified people at risk of developing a vitamin D deficiency; however, only a few (3%) knew that people with dark skin can be at greater risk of developing vitamin D deficiency. Similarly, almost one-third were aware that the use of sunscreen and time of day might affect vitamin D synthesis; however, only a few recognized other factors that affect vitamin D synthesis, including seasons (11.7%), cloud cover (16.5%), skin pigmentation (12%), latitude (3%), and pollution (10%). Furthermore, a small proportion of participants specified a high-fat diet (18.6%) and smoking (8.7%) as factors affecting vitamin D synthesis. The majority of health educators believed that vitamin D helps in the absorption of calcium in the body (76%) and that the intake of vitamin D supplements helps reduce the risk of vitamin D deficiency (90%). The most frequent sources of information regarding vitamin D among study participants were doctors (64%), family/friends (50%), the internet (48.5%), and television (32.5%). Overall, 45% of health educators had good knowledge of vitamin D.

### 3.3. Attitudes Regarding Vitamin D

Health educators’ attitudes regarding vitamin D questions and responses are presented in Table 3. Only 29% of health educators agreed that they must expose themselves to sunlight. However, almost all participants believed that vitamin D is important for health. Approximately one-third of health educators believed that sunlight might damage the skin, while 41% disagreed with this belief. Additionally, 67% of study participants were concerned about their vitamin D levels, and almost all participants were willing to undergo vitamin D tests if needed. Overall, 42.9% of health educators had a positive attitude towards vitamin D.

### 3.4. Factors Associated with Knowledge of and Attitudes towards Vitamin D

**Figure 1:**
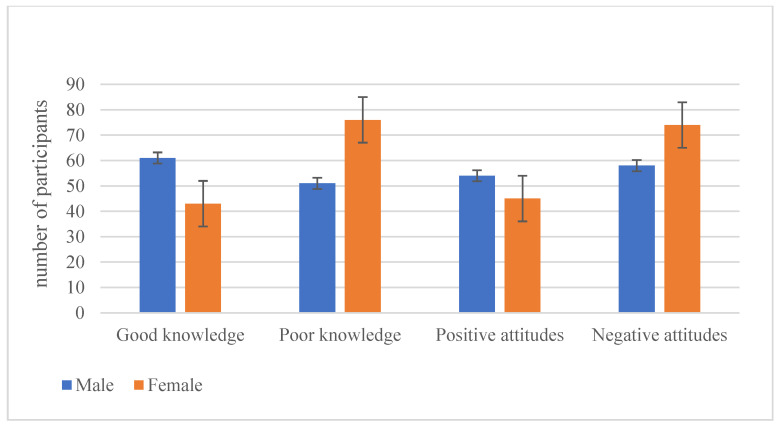
Gender differences in knowledge and attitudes toward vitamin D among health educators in public schools.

Table 4 presents the sociodemographic characteristics associated with health educators’ knowledge of and attitudes towards vitamin D. Male health educators were found to be more knowledgeable than female health educators (58.7% and 41.3%, respectively; *p* = 0.005) (Figure 1). In addition, health educators with bachelor’s degrees (74%) were more knowledgeable than diploma (21.2%) and postgraduate holders (4.8%). Moreover, male health educators had positive attitudes toward vitamin D more than female health educators; however, the finding was not statically significant (Figure 1). Overall, only 45% of health educators in the current study had good knowledge of vitamin D, and approximately 43% had positive attitudes towards vitamin D. Table 5 presents the association between the scores for knowledge of and attitude towards vitamin D and selected sociodemographic factors for health educators stratified by gender. 

Attitude scores indicated a positive association with age among male health educators (*p* = 0.007). Male health educators aged 45–54 years had the highest scores for attitude (3.8 ± 0.7) compared to male health educators in other age groups. However, there was no significant association of age with knowledge and attitude scores among female health educators. Further, marital status also showed a positive association with attitude scores among female health educators (*p* = 0.04). Divorced and widowed females had higher scores for attitude (3.8 ± 0.7 and 3.6 ± 0.5, respectively) compared to single and married females (3.1 ± 0.6 and 3.2 ± 0.7, respectively). However, marital status did not show any significant association with knowledge and attitude scores among male health educators.

Knowledge scores showed a positive association with educational levels among male health educators (*p* = 0.001). Male health educators with postgraduate degrees had the highest knowledge scores (13.5 ± 4.6) compared to those holding diplomas and bachelor’s degrees (9.9 ± 2.6 and 12.16 ± 3.3, respectively). However, educational level did not have a significant association with knowledge and attitude scores among female health educators.

Additionally, female health educators who were administrators had the highest attitude scores (3.3 ± 0.7) compared to female health educators who were teachers (3 ± 0.6) (*p* = 0.01). Furthermore, school stage, work experience, and specialization did not exhibit any significant association with knowledge and attitude scores among either male or female health educators.

## 4. Discussion

Vitamin D deficiency is a key health issue worldwide. It is highly prevalent in Saudi Arabia among all age groups; however, it is more prevalent among females and children. Health educators play vital roles in enhancing health awareness for future generations in schools, including expanding the awareness of the importance of vitamin D and the effects of vitamin D deficiency on human health; thus, it is essential for health educators in schools to have good knowledge of and a positive attitude towards vitamin D. The aim of this study was to examine the knowledge of and attitude towards vitamin D among health educators in public schools in Jeddah. The findings of the current study revealed that only 45% of health educators had good knowledge of vitamin D, and approximately 43% had positive attitudes towards vitamin D. Additionally, those who had good knowledge of vitamin D were males who had bachelor’s and postgraduate degrees. Similarly, male health educators aged 45–54 years had positive attitudes towards vitamin D. In addition, female health educators who were divorced and widowed and those who were administrators had positive attitudes towards vitamin D.

Overall, 45% of health educators in the current study had a good knowledge of vitamin D; however, most of the study participants indicated that they were aware of vitamin D and its importance. In fact, the proportion of those with a good knowledge of vitamin D (45%) among health educators in public schools is considered insufficient in terms of how important it is to communicate such knowledge to students and teachers. However, other studies among different populations, including the general population and university students within Saudi Arabia have reported inconsistent findings [23,30,31]. For example, a study that examined the knowledge of vitamin D among the general population in Jeddah, Saudi Arabia, reported the overall knowledge at approximately 39% [23], which is lower than the rate reported in our study. In contrast, a study conducted among the general population in Al-Baha city, Saudi Arabia, reported that 69.5% of study participants had adequate knowledge of vitamin D [30]. Similarly, a study conducted among university students in Al-Kharj city, Saudi Arabia, reported that 85% of study participants had a good knowledge of vitamin D [31]. However, no previous studies have examined the knowledge of and attitude towards vitamin D among health educators in schools in Saudi Arabia.

Furthermore, the results of the current study revealed that the most common sources of information regarding vitamin D in this study were doctors (who are the primary source of knowledge), then family and friends, and then the internet; this is consistent with a study in France [32] but contrasting with a study in England, where the media was the most common source of knowledge about vitamin D [33]. Similarly, in Saudi Arabia, another study reported that the first source of information about vitamin D was relatives and friends (56%), followed by healthcare professionals (51%), while the internet was the primary source of information among 46% of the study participants [34]. In terms of the knowledge of sources of vitamin D (exposure to sunlight, diet, and supplements), a study in the UK reported that 99% of participants were aware that exposure to sunlight is a good source of vitamin D, compared to only 49% of participants who were aware of this in the current study. Similarly, in the UK study, the majority of study participants had better knowledge regarding diet (84% vs. 30%) and supplements (87% vs. 27%) as good sources of vitamin D compared to the participants of this study [33]. Further, a study conducted among Saudis living in Jeddah city reported that 86% of participants were aware that sun exposure is a good source of vitamin D, and only 21% were aware that vegetables and fruits are poor dietary sources of vitamin D, which reflects slightly better knowledge of vitamin D sources than that reported in our study [23]. Additionally, in a study conducted in Riyadh, Saudi Arabia, 98% of the participants were aware that sunlight is the main source of vitamin D, 92% mentioned vitamin D supplements, 57% mentioned fatty fish, 43% mentioned milk, 35% mentioned eggs, 56% mentioned fruits, and 35% mentioned vegetables as sources of vitamin D [35]. Similarly, in the recent study conducted in Khartoum in Sudan on the knowledge of vitamin D deficiency among health professionals, 71% of the participants were aware that direct skin exposure to sunlight is necessary for the production of vitamin D [36]. In addition, in a study among coronary heart patients in Saudi Arabia, only a few participants were aware of the dietary sources of vitamin D, such as milk (4% of heart patients and 10% of controls) and fatty fish (11% of heart patients and 25% of controls) [27].

Furthermore, in this study, almost 95% of study participants were aware of the traditional role of vitamin D in strengthening bones and bone health, and 76% were aware that vitamin D helps in the absorption of calcium in the body; however, only 25% were aware of the role of vitamin D in protecting against heart disease. In fact, recent research has indicated that vitamin D deficiency is associated with a risk of cardiovascular disease, plays a protective role against cardiovascular disease and associated risk factors, and reduces the risk of heart failure [37]. Furthermore, in a study on coronary heart disease patients in Saudi Arabia, 46% of the patients also had a vitamin D deficiency [27]. Additionally, a study conducted in Saudi Arabia among the female population revealed that only 20% of participants were aware that vitamin D is beneficial in protecting against heart disease [34]. This percentage is less than that reported in our study, which might be due to the high level of education among the participants of our study. Additionally, it is common to be educated about the traditional role of vitamin D in bone health by media sources, by family or friends, or by way of advice from doctors. This enables the creation of a link between vitamin D and bone health in the minds of people. Furthermore, a previous study revealed that 68% of Saudi females had good knowledge of the benefits of vitamin D for bone health [34]. Similarly, previous studies conducted in Saudi Arabia and the UK revealed that 69% and 82% of participants, respectively, were aware of the role of vitamin D in bone health [23,32,33]. However, lower levels of knowledge regarding vitamin D were demonstrated in other areas, including people at risk of developing vitamin D deficiency and factors affecting vitamin D synthesis from sunlight. Almost one-fourth of the participants were aware that individuals who do not go outdoors in the sunlight or cover their skin when out under the sun might develop vitamin D deficiency, while only 3% were aware that individuals with dark skin are more likely to develop vitamin D deficiency as compared to people with lighter skin. In contrast, 90% of the participants were aware of the importance of taking vitamin D supplements to prevent vitamin D deficiency. Moreover, health educators were aware that vitamin D is created in the body, and almost one-third were aware of the effect of the time of day and the use of sunscreen in preventing the synthesis of vitamin D. However, a few participants identified other factors that affect vitamin D synthesis from sunlight, such as seasons, skin pigmentation, cloud cover and latitudes, and almost one-quarter of health educators reported that they do not know about any factors that affect vitamin D synthesis. The abovementioned results are consistent with a previous study in Iran, where only 16% of the participants were aware that a lack of exposure to sunlight influenced the vitamin D levels in the body [38]. Similarly, a study in Saudi Arabia showed that 26% of participants knew the effect of using sunscreen, and 13% of them knew that skin colour is among the factors affecting vitamin D synthesis [39]. However, a study conducted in the UK revealed better knowledge among participants, as participants had fairly good knowledge of the influence of certain factors on vitamin D synthesis, including skin pigmentation (58%), use of sunscreen (55%), and time of day (49%) [33].

With regard to health educators’ attitudes towards vitamin D, only 43% of health educators in this study had positive attitudes towards vitamin D. However, almost all health educators admitted the importance of vitamin D and agreed to test for the serum levels of vitamin D in their bodies if their health condition required it. Similarly, two-thirds were concerned about having vitamin D deficiency. In contrast, only 29% of health educators agreed to expose themselves to sunlight regularly. Similarly, almost 60% of health educators believed that exposure to sunlight is harmful to the skin or were nonaligned with this belief. This attitude towards exposure to sunlight might be the reason for the high prevalence of vitamin D deficiency in the Kingdom. Another recent study conducted among pre-menopausal women in Jeddah, Saudi Arabia, reported that approximately 50% of study participants did not like to expose themselves to sunlight [39]. These findings were in agreement with a study conducted in Malaysia, where 53% of the participants avoided exposure to sunlight [29]. In contrast, a previous study in the UK reported that over half of the population prefers exposure to sunlight [33]. Avoiding sun exposure could be attributed to the geographical location of Saudi Arabia, where there is scorching sunlight all year, as opposed to Northern Europe. Additionally, two-thirds of health educators were concerned that their vitamin D levels were too low, which is consistent with a previous study in the UK in which half of the participants were concerned that their vitamin D levels were deficient [33].

With regards to the sociodemographic factors associated with knowledge of and attitude towards vitamin D, there were significant differences in the knowledge of vitamin D between males and females, as 59% of males had good knowledge of vitamin D compared to 41% of females. A previous study in Riyadh, Saudi Arabia, reported similar results, where 54% of males had good knowledge of vitamin D compared with 49% of females with such knowledge; however, the result was not statistically significant [40]. This is probably because the majority of university degree holders (bachelor’s and postgraduate) in this study were males and not females. Moreover, our results indicate that health educators with university degrees had good knowledge of vitamin D; more specifically, male health educators with bachelor’s and postgraduate degrees had significantly higher knowledge of vitamin D than other health educators. These findings are consistent with a previous study conducted in Saudi Arabia that revealed that the level of knowledge was significantly associated with higher educational levels, where bachelor’s and postgraduate degree holders had higher knowledge of vitamin D than others [23]. Furthermore, the scores for attitude towards vitamin D were significantly associated with age among male health educators only, as those aged 45–54 years had higher scores of attitudes towards vitamin D than other age groups. This might be because this age group is considered as ‘seniors’, and they are more likely to suffer from various diseases and tend to follow doctors’ instructions to maintain good health [41]; thus, they usually have positive attitudes towards maintaining a healthy lifestyle. In fact, a study conducted in the UK indicated that nutritional knowledge increased among middle-aged people and decreased significantly among younger and older people, which indicates that middle-aged people are more knowledgeable regarding nutritional aspects and, therefore, tend to adopt a positive attitude towards a healthy lifestyle [41]. Higher scores of attitudes towards vitamin D among female health educators were significantly associated with being divorced or widowed. In a study conducted among females in Saudi Arabia, widows had the highest degree of vitamin D practices [34], which is slightly similar to our results. This might be because widowed and divorced women bear more responsibility for their health and the health of their children and families than married women who receive support from their husbands; hence, widowed and divorced women are more likely to have positive attitudes towards following health instructions. Additionally, female health educators who work as administrators had higher scores for attitudes vitamin D than those who work as teachers. However, the reason for such a difference may be the small number of teachers who participated in the current study.

To the best of our knowledge, this is the first study to examine the knowledge of and attitude towards vitamin D among health educators in public schools in Saudi Arabia. Health educators in schools are neglected in terms of scientific research and health education, even though they are a very important and integral part of the education system, as health educators promote community health through their communication with teachers and parents. Consequently, they have a great influence in transferring knowledge, imparting scientific health concepts, and correcting misconceptions among students, teachers, and parents. Therefore, the findings of this study provide the MOE in the Kingdom with recommendations to enhance the roles of health educators and enhance the quality of health education provided in schools. Nevertheless, the current study has several limitations. First, this was a cross-sectional study, as it only demonstrated an association. Second, it is possible that selection bias was introduced when collecting data via a self-administered online questionnaire; however, validated questionnaires were used for data collection. Third, data regarding vitamin D practices were not collected from health educators, which will reflect the actual vitamin D-related behaviour such as exposure to sunlight, and the intake of vitamin D supplements and diet rich of vitamin D among this group; we recommend that future studies bridge this gap. Fourth, because serum vitamin D was not collected from study participants, correlations of knowledge of and attitudes towards vitamin D with serum vitamin D levels were not determined. Last, it may not be possible to generalise the findings of the current study to all health educators in the Kingdom; however, the findings are believed to be representative of health educators in Jeddah city, as health educators who participated in this study were recruited from the six educational administrative offices in Jeddah.

## 5. Conclusions

The results of the current study revealed that over half of the health educators in Jeddah city had poor knowledge and negative attitudes towards vitamin D, which is insufficient when considered against the background their significant role in improving health awareness among future generations, including enhancing awareness of the importance of vitamin D and the effects of the deficiency of vitamin D on human health. The MOE must consider implementing educational programmes for health educators in public schools to improve their knowledge regarding vitamin D, particularly among female health educators and in aspects such as sources of vitamin D and individuals at risk of developing vitamin D deficiency. Additionally, educational programmes should emphasize the importance of exposure to sunlight as the main source of vitamin D and assure that sunlight exposure is a safe and healthy practice, particularly during certain specific times of the day, which will help to establish positive attitudes towards vitamin D and sun exposure among this significant group. Furthermore, future studies should examine vitamin D-related behaviour among health educators and investigate the correlations among knowledge of and attitude and behaviour towards vitamin D and serum vitamin D levels among health educators in schools. In addition, it is important to conduct qualitative research in the future to uncover the reasons for poor knowledge and a negative attitude towards vitamin D, which have possibly influenced the high prevalence of vitamin D deficiency in the population in Saudi Arabia.

## Figures and Tables

**Table 1:** Socio-demographic characteristics of health educators stratified by gender.

Characterization	Total (n = 231) n (%)	Male (n = 112) n (%)	Female (n = 119) n (%)	*p* Value
Age				
<34	26 (11.3)	21 (18.8)	5 (4.2)	<0.001
35–44	138 (59.7)	69 (61.6)	69 (58)	
45–54	58 (25.1)	19 (17)	39 (32.8)	
55–62	9 (3.9)	3 (2.7)	6 (5)	
Marital status				
Single	17 (7.4)	10 (8.9)	7 (5.9)	0.05
Married	194 (84)	97 (86.6)	97 (81.5)	
Divorced	14 (6.1)	5 (4.5)	9 (7.6)	
Widow	6 (2.6)	0	6 (5)	
Education level				
Diploma	66 (28.6)	26 (23.2)	40 (33.6)	0.16
Bachelor	159 (68.8)	82 (73.2)	77 (64.7)	
Postgraduate	6 (2.6)	4 (3.6)	2 (1.7)	
School stage				
Primary	103 (44.6)	35 (31.3)	68 (57.1)	<0.001
Intermediate	65 (28.1)	34 (30.4)	31 (26.1)	
Secondary	63 (27.3)	43 (38.4)	20 (16.8)	
Experience years				
1–5	30 (13)	11 (9.8)	19 (16)	<0.001
6–10	87 (73.7)	21 (18.8)	66 (55.5)	
11–15	46 (19.9)	32 (28.6)	14 (11.8)	
16–20	33 (14.3)	28 (25)	5 (4.2)	
Above 20	35 (15.2)	20 (17.9)	15 (12.6)	
Educational offices				
North	59 (25.5)	8 (7.1)	51 (42.9)	<0.001
East	74 (32)	46 (41.1)	28 (23.5)	
Centre	36 (15.6)	27 (24.1)	10 (8.4)	
South	42 (18.2)	12 (10.7)	30 (25.2)	
Naseem	11 (4.8)	11 (9.8)	0	
Safa	9 (3.9)	8 (7.1)	0	
Job title				
Administrator	168 (72.7)	77 (68.8)	91 (76.5)	0.18
Teacher	63 (27.3)	35 (31.3)	28 (23.5)	
Specialization				
Islamic	36 (15.6)	24 (21.4)	12 (10)	0.004
Linguistic	36 (15.6)	17 (15.2)	19 (16)	
Science	53 (22.9)	26 (23.2)	27 (22.7)	
Computer science	5 (2.2)	3 (2.7)	2 (1.7)	
Home Economics and Art	19 (8.2)	14 (12.5)	5 (4.2)	
Social Science	31 (13.4)	16 (14.3)	15 (12.6)	
Management and Economics	26 (11.3)	5 (4.5)	21 (17.6)	
Media	3 (1.3)	0	3 (2.5)	
General	16 (6.9)	5 (4.5)	11 (9.2)	
No speciality	6 (2.6)	2 (1.8)	4 (3.4)	

Chi square test used to examine difference between two groups; *p* value of 0.05 was considered statistically significant.

**Table 2:** Health educator’ knowledge regarding vitamin D.

Knowledge Questions	Total (n = 231) n (%)
Have you ever heard of Vitamin D?	
Yes	230 (99.6)
No	1 (0.4)
Do you think Vitamin D is important for your health?	
Yes	229 (99.1)
No	0
I don’t know	2 (0.9)
Where did you hear about Vitamin D or Vitamin D deficiency?	
Doctor	148 (64.1)
Nurse	19 (8.2)
Internet	112 (48.5)
Family/Friends	117 (50.6)
Pharmacist	19 (8.2)
Newspaper	36 (15.6)
Television	75 (32.5)
Other	5 (2.2)
Where do you think the body gets vitamin D from?	
Diet	70 (30.3)
Sun exposure	114 (49.4)
supplements	62 (26.8)
I don’t know	1 (0.4)
All of the above	155 (67.1)
What type of food is a good source of vitamin D?	
Vegetables & Fruits	71 (30.7)
Milk	80 (34.6)
Fatty fish (salmon, sardine)	162 (70.1)
Olive oil	25 (10.8)
Egg	84 (36.4)
I don’t know	19 (8.2)
Which of the following you think are the benefits of Vitamin D?	
Strong bones	218 (94.4)
Prevents heart diseases	62 (26.8)
It has no benefit	0
I don’t know	6 (2.6)
Vision	36 (15.6)
Prevents anemia	39 (16.9)
In your opinion which one of the following categories is more at risk of developing Vitamin D deficiency?	
Individuals not outdoors often	63 (27.3)
Cover up skin when out	46 (19.9)
Individuals with dark skin	7 (3)
Individuals who avoid sun exposure	202 (87.4)
None of the above	2 (0.9)
I don’t know	9 (3.9)
Vitamin D is synthesized inside our body?	
Yes	172 (74.5)
No	29 (12.6)
I don’t know	30 (13)
Factors affecting vitamin D synthesis from sunlight.	
Season	27 (11.7)
Skin Pigmentation	28 (12.1)
Sunscreen use	72 (31.2)
Time of day	75 (32.5)
Cloud cover	38 (16.5)
Latitude	7 (3)
Pollution	23 (10)
Smoking	20 (8.7)
High-fat diet	43 (18.6)
None of the above	13 (5.6)
I don’t know	58 (25.1)
Vitamin D helps the absorption of calcium in the body?	
Yes	175 (75.8)
No	10 (4.3)
I don’t know	46 (19.9)
Taking Vitamin D supplements reduces the risk of Vitamin D deficiency	
Yes	208 (90)
No	6 (2.6)
I don’t know	17 (7.4)

**Table 3:** Health educator’ attitudes regarding vitamin D.

Attitudes Questions	Total (n = 231) n (%)
I like to expose myself to sunlight	
Agree	67 (29)
Disagree	91 (39.4)
Neither agree or disagree	73 (31.6)
Do you think vitamin D is important for your health?	
Yes	229 (99.1)
No	0
I don’t know	2 (0.9)
The exposure to sunlight is harmful for the skin.	
Agree	81 (35.1)
Disagree	94 (40.7)
Neither agree or disagree	56 (24.2)
I am concerned that my current vitamin D levels might be too low.	
Agree	154 (66.7)
Disagree	57 (24.7)
Neither agree or disagree	20 (8.7)
I am willing to undergo test for vitamin D if a medical condition demands it	
Yes	230 (99.6)
No	1 (0.4)

**Table 4:** Socio-demographics associated with health educators’ knowledge and attitudes toward vitamin D.

Socio-Demograhics	Knowledge		*p* Value	Attitudes		*p* Value
	Good Knowledge n = 104 n (%)	Low Knowledge n = 127 n (%)		Positive Attitudes n = 99 n (%)	Negative Attitudes n = 132 n (%)	
Age						
<34	13 (12.5)	13 (10.2)	0.76	16 (16.2)	10 (7.6)	0.06
35–44	60 (57.7)	78 (61.4)		53 (53.5)	85 (64.4)	
45–54	28 (26.9)	30 (23.6)		28 (28.3)	30 (22.7)	
55–62	3 (2.9)	6 (4.7)		2 (2)	7 (5.3)	
Gender						
Male	61 (58.7)	51 (40.2)	0.005	54 (54.5)	58 (43.9)	0.11
Female	43 (41.3)	76 (59.8)		45 (45.5)	74 (56.1)	
Marital status						
Single	6 (5.8)	11 (8.7)	0.76	7 (7.1)	10 (7.6)	0.61
Married	90 (86.5)	104 (81.9)		81 (81.8)	113 (85.6)	
Divorced	6 (5.8)	8 (6.3)		7 (7.1)	7 (5.3)	
Widow	2 (1.9)	4 (3.1)		4 (4)	2 (1.5)	
Education level						
Diploma	22 (21.2)	44 (34.6)	0.01	30 (30.3)	36 (27.3)	0.8
Bachelor	77 (74)	82 (64.6)		67 (67.7)	92 (69.7)	
Postgraduate	5 (4.8)	1 (0.8)		2 (2)	4 (3)	
School stage						
Primary	43 (41.3)	60 (47.2)	0.52	44 (44.4)	59 (44.7)	0.94
Intermediate	29 (27.9)	36 (28.3)		27 (27.3)	38 (28.8)	
Secondary	32 (30.8)	31 (24.4)		28 (28.3)	35 (26.5)	
Experience years						
1–5	13 (12.5)	17 (13.4)	0.26	10 (10.1)	20 (15.2)	0.59
6–10	33 (31.7)	54 (42.5)		36 (36.4)	51 (38.6)	
11–15	21 (20.2)	25 (19.7)		22 (22.2)	24 (18.2)	
16–20	20 (19.2)	13 (10.2)		17 (17.2)	16 (12.1)	
Above 20	17 (16.3)	18 (14.2)		14 (14.1)	21 (15.9)	
Job title						
Administrator	74 (71.2)	94 (74)	0.62	76 (76.8)	92 (69.7)	0.23
Teacher	30 (28.8)	33 (26)		23 (23.2)	40 (30.3)	
Specialization						
Islamic	21 (20.2)	15 (11.8)	0.18	18 (18.2)	18 (13.6)	0.8
Linguistic	12 (11.5)	24 (18.9)		17 (17.2)	19 (14.4)	
Science	24 (23.1)	29 (22.8)		20 (20.2)	33 (25)	
Computer science	3 (2.9)	2 (1.6)		3 (3)	2 (1.5)	
Home Economics and Art	6 (5.8)	13 (10.2)		7 (7.1)	12 (9.1)	
Social Science	16 (15.4)	15 (11.8)		14 (14.1)	17 (12.9)	
Management and Economics	9 (8.7)	17 (13.4)		8 (8.1)	18 (13.6)	
Media	0	3 (2.4)		2 (2)	1 (0.8)	
General	9 (8.7)	7 (5.5)		8 (8.1)	8 (6.1)	
Not specified	4 (3.8)	2 (1.6)		2 (2)	4 (3)	

Chi square test used to examine difference between two groups; *p* value of 0.05 was considered statistically significant.

**Table 5:** Association between knowledge, and attitude scores toward vitamin D and selected socio-demographics for health educators stratified by gender.

Socio-Demographics	Male		Female	
	Knowledge	Attitude	Knowledge	Attitude
Age				
<34	11.8 ± 3.1	3.6 ± 0.7	8.8 ± 2.7	3.6 ± 0.5
35–44	12.1 ± 3.4	3.2 ± 0.8	10.4 ± 3.1	3.3 ± 0.7
45–54	11.5 ± 3.6	3.8 ± 0.7	11.1 ± 2.6	3.2 ± 0.7
55–62	14 ± 5.2	2.6 ± 0.5	9.8 ± 1.9	3.1 ± 0.7
*p* value	0.7	0.007	0.29	0.7
Marital status				
Single	10.9 ± 2.6	3.6 ± 0.6	11.5 ± 3.9	3.1 ± 0.6
Married	12.1 ± 3.5	3.4 ± 0.8	10.7 ± 2.7	3.2 ± 0.7
Divorced	12.4 ± 3.2	2.6 ± 1.5	8.6 ± 2.9	3.8 ± 0.7
Widow	0		10.5 ± 3.6	3.6 ± 0.5
*p* value	0.54	0.08	0.18	0.04
Education level				
Diploma	9.9 ± 2.6	3.5 ± 0.8	10.16 ± 3.2	3.3 ± 0.7
Bachelor	12.16 ± 3.3	3.3 ± 0.8	10.4 ± 2.7	3.62 ± 0.7
Postgraduate	13.5 ± 4.6	3.2 ± 0.5	14 ± 1.4	3.5 ± 2.1
*p* value	0.001	0.63	0.25	0.68
Job title				
Administrator	11.6 ± 2.9	3.3 ± 0.8	10.4 ± 2.9	3.3 ± 0.7
Teacher	12.8 ± 4.2	3.4 ± 0.8	11 ± 2.8	3 ± 0.6
*p* value	0.08	0.49	0.37	0.01

Linear regression was used to examine the association between sociodemographic characteristics and knowledge of and attitude towards vitamin D scores; *p* value of 0.05 was considered statistically significant.

## Data Availability

The datasets generated and/or analyzed during this study are not publicly available owing to use of data for further publications but are available from the corresponding author on reasonable request.

## Supplementary Materials

The following supporting information can be downloaded at: https://www.mdpi.com/article/10.3390/healthcare10122358/s1.
