# Supplementary material for: Knowledge and Attitudes towards Vitamin D among Health Educators in Public Schools in Jeddah, Saudi Arabia: A Cross-Sectional Study
Source: Healthcare (Basel). 2022 Nov 24;10(12):2358. doi: 10.3390/healthcare10122358 (PMC9777692; doi:10.3390/healthcare10122358)
Supplement: Supplementary file 1 [file healthcare-10-02358-s001.zip › healthcare-1981255-supplementary.pdf]

Supplementary File

1 – Socio-demographic characteristics of the health educator:

1 .Gender: □ Male □ Female

2 .Age in years: □ 20- 24 □ 25-34 □ 35-44 □ 45-54 □ 55-62

3- Social status: □ Single □ Married □ divorced □ widow.

4- Qualification: □ diploma □ Bachelor □ Masters or PhD

5- Specialization: ___________________________

6- Job title: □ Teacher □ Managerial

7- School level: □ elementary □ intermediate □ secondary

8- Education Office: □ North □ Middle □ East □ South □ Al-Safa □ Al-Naseem

9- Years of Experience: □ 1-5 □ 6-10 □ 11-15 □ 16-20 □ 21 and more

2 - Participant's knowledge of vitamin D:

1 .Have you ever heard of Vitamin D?

□ Yes □ No

2 .Do you think Vitamin D is important for your health?

□ Yes □ No □ I don't know

3 .Where did you hear about Vitamin D or Vitamin D deficiency?

□ Doctor □ Nurse □ Internet □ Family/Friends

□ Pharmacist □ Newspaper □ Television □ Other (specify)__________

4 .Where do you think the body gets vitamin D from?

□ Diet □Sun exposure □ supplements

□ I don't know □ All of the above

5- What type of food is a good source of vitamin D?

□Vegetables & Fruits □Milk □ Fatty fish (salmon, sardine)

□ Olive oil □ Egg □ I don’t know

6- Which of the following you think are the benefits of Vitamin D?

□ Strong bones □ Prevents heart diseases □ It has no benefit

□ I don’t know □ Vision □ Prevents anemia

7- In your opinion which one of the following categories is more at risk of developing Vitamin D deficiency?

□ Individuals not outdoors often □ Cover up skin when out

□ Individuals with dark skin □ Individuals who avoid sun exposure

□ None of the above □ I don’t know

8- Vitamin D is synthesized inside our body?

□ Yes □ No □ I don't know

9- Factors affecting vitamin D synthesis from sunlight?

□ Season □ Skin Pigmentation □ Sunscreen use □ Time of day

□ Cloud cover □ Latitude □ Pollution □ Smoking

□ High-fat diet □ None of the above □ I don’t know

10- Vitamin D helps the absorption of calcium in the body?

□ Yes □ No □ I don't know

11- Taking Vitamin D supplements reduces the risk of Vitamin D deficiency

□ Yes □ No □ I don't know

3 - Participant's attitude to vitamin D:

1- I like to expose to sunlight all the time.

□ Disagree □ Neither agree or disagree □ Agree

2- Do you think vitamin D is important for your health?

□ Yes □ No □ I don't know

3- The exposure to sunlight is harmful for the skin.

□ Disagree □ Neither agree or disagree □ Agree

4- I am concerned that my current vitamin D levels might be too low.

□ Disagree □ Neither agree or disagree □ Agree

5- I am willing to undergo test for vitamin D if a medical condition demands it.

□ Yes □ No

End
